# Characterizing Fidelity Monitoring Practices in Community Behavioral Health Care

**DOI:** 10.1007/s11414-025-09967-x

**Published:** 2025-09-10

**Authors:** Brigid R. Marriott, Allison E. Meyer, Amanda Feagans, Brielle L. Batch, Zachary W. Adams

**Affiliations:** 1 Adolescent Behavioral Health Research Program, Indiana University School of Medicine, Indianapolis, IN USA; 2 Department of Psychiatry, Indiana University School of Medicine, Indianapolis, IN USA

## Abstract

Evidence-based practices (EBPs) are most effective when they are delivered with a high degree of fidelity, or as they are intended to be delivered. Because clinicians often deviate from fidelity, it is important to monitor EBP fidelity over time to guide corrective actions. However, little is known about current fidelity monitoring practices in community behavioral health care. The current study used a mixed methods approach to characterize current fidelity monitoring practices, as well as barriers and facilitators to fidelity monitoring, in community behavioral health care agencies. Therapists, supervisors, recovery coaches, executive leaders, and agency leaders (*N* = 191) from multiple agencies in a Midwestern state completed a survey measuring current fidelity monitoring methods at their agency and perceived acceptability and feasibility of potential fidelity monitoring methods and strategies. Additionally, agency leaders, supervisors, and therapists (*N* = 10) within the state and leaders of intermediary organizations (*N* = 11) across the United States participated in individual qualitative interviews asking about facilitators, barriers, and priorities related to ongoing fidelity monitoring. Most respondents indicated their agency currently monitors what practices are being delivered, with self-report and chart review the most frequently reported methods used and session recordings and role-play assessment the least-frequently endorsed. Mixed methods results revealed common barriers to and potential strategies for facilitating fidelity monitoring efforts at the clinician-, agency-, and system-level. Findings highlight the need for scalable and sustainable methods for monitoring fidelity and the need for multi-level approaches to support EBP fidelity monitoring in community behavioral health settings.

## Introduction

Nearly 20% of the US population experiences a mental health or substance use disorder each year.^1,2^ Multiple behavioral health evidence-based practices (EBPs) have been developed to effectively treat a variety of behavioral and mental health conditions across the lifespan.^3–5^ Behavioral health EBPs refer to services that are guided by best practices, clinical expertise, characteristics of the patient population, and empirical research.^6^ Such EBPs result in better clinical outcomes than treatment as usual^5^ and, over time, yield organizational cost-savings.^7^ Despite these well-established benefits, EBPs are inconsistently available.^8^ To address these gaps, there have been numerous investments at the local, state, and federal levels to expand dissemination of EBPs through training and implementation support.^9,10^

When EBPs are available, these interventions may not be delivered with fidelity, that is, as intended.^11,12^ As a result, services may suffer from program drift, in which deviating from EBP fidelity yields reduced clinical benefit of the intervention for patients.^13^ Moreover, because EBP implementation can be costly,^14^ organizations need to ensure that there is an adequate return on their investment to sustain their use, and low EBP fidelity may jeopardize these investments.^15^ While some studies have not found that higher fidelity results in better treatment outcomes,^16^ most research indicates EBPs are most effective when they are delivered with a high degree of fidelity.^17–22^ Therefore, it is important to monitor EBP fidelity over time to ensure clinicians are delivering EBPs as intended and to minimize program drift, as well as to guide corrective actions to improve fidelity.

Agencies may use different approaches to train, implement, and monitor fidelity to EBPs within their sites. For example, agencies may leverage internal agency support (e.g., learning materials, supervision) or partner with an external entity, such as intermediary organizations (IOs), which provide agencies with assistance and support to build capacity to select, implement, and sustain EBPs within their agency.^23^ It is likely that fidelity monitoring approaches may differ depending on whether fidelity monitoring is being managed internally or externally. In addition, the fidelity monitoring methods deemed feasible and acceptable within an agency may vary depending on an individual’s role (e.g., clinician, supervisor, agency leader, IO leader). For instance, one study found clinicians were most concerned about organizational support, the scope of their clinical practice, and the time-related resources needed to implement and sustain fidelity monitoring practices.^24^ While agency leader’s views on feasible fidelity monitoring practices have received less attention, similar concerns as those associated with EBP implementation and sustainment, such as costs, may be present for agency leaders.^25^ IO leaders may hold a third viewpoint, whereby their concerns may largely be on leveraging strategies, such as identifying a fidelity monitoring champion, to implement and sustain fidelity monitoring practices over time.^26^

Further, the acceptability and feasibility of different fidelity monitoring methods is likely to be impacted by available resources. Direct observation of a clinician’s sessions, where trained personnel review and code a live or recorded session for competence and adherence to an EBP, is considered the gold standard for fidelity monitoring for many EBPs.^27^ However, direct observation presents several resource-related barriers including cost, labor, and time constraints.^28,29^ To address these barriers, alternative fidelity monitoring methods have been utilized, such as behavioral-rehearsal or role-play assessment,^30,31^ chart-stimulated recall,^32^ and clinician self-report using a fidelity checklist.^33^ A study designed to compare fidelity monitoring methods to the gold standard of observational coding found behavioral-rehearsal to be comparable to observational coding, but self-report and chart-stimulated recall both overestimated fidelity.^34^ Thus, available resources (e.g., recording equipment, time for reviewing recorded sessions) may influence the fidelity monitoring method used.

Little is known about current fidelity monitoring practices in community behavioral health settings.^35,36^ Given the cost-, time-, and resource-related barriers to using direct observation, it is unlikely that community behavioral health settings have the capacity (e.g., administrative support, time, staff) to frequently engage in the gold-standard fidelity monitoring procedures.^28,29^ Instead, community behavioral health settings may be using alternative approaches to monitoring fidelity or assessing the quality of mental health care being delivered. There is a need for data on the fidelity monitoring practices already taking place in community behavioral health settings, as well as on what fidelity monitoring procedures are considered feasible, particularly within a state facing challenges with workforce shortages, limited funding, and high administrative burden like Indiana.^37^ A clearer understanding of current fidelity monitoring practices and attitudes may provide a basis for advancing practical, effective, and sustainable fidelity monitoring efforts.

Thus, the purpose of this study was (a) to characterize current fidelity monitoring practices in community behavioral health care in Indiana and (b) to better understand what fidelity monitoring approaches are seen as acceptable and feasible by the behavioral health workforce. This project arose from a service contract with the state of Indiana to develop an infrastructure, guided by local behavioral health partners’ input, for ongoing EBP fidelity monitoring. In addition to state-level behavioral health partners, this project sought input from IO partners across the United States. Using a mixed methods approach, the authors distributed surveys to behavioral health professionals (therapists, supervisors, recovery coaches, executive leaders, and agency leaders) across Indiana and conducted interviews with Indiana behavioral health clinicians, supervisors, and agency leaders as well as IO leaders.

## Methods

### Procedure

The current study uses data from a project that arose from a service contract with the state of Indiana’s Division of Mental Health & Addiction (DMHA) to develop partner-guided infrastructure and procedures for ongoing behavioral health EBP fidelity monitoring. As a first step in the service project, a simultaneous mixed methods approach was used to gather input from behavioral health (BH) professionals and agency leaders in the state and leaders of IOs in other states. The quantitative data were collected using a web-based survey administered to individuals who worked in the behavioral health field in Indiana. The qualitative data entailed individual semi-structured interviews conducted with BH clinicians, supervisors, and agency leaders in Indiana and IO leaders in other states. The current study received the necessary approvals from Indiana University’s Institutional Review Board. We followed the STROBE reporting guidelines for observational studies.

#### Quantitative

An email with a link to a secure, web-based survey was sent to potential participants by the sponsoring mental health agency’s communication team to relevant listservs they maintained as well as to counselors and therapists who had previously participated in the authors’ behavioral health continuing education programs and EBP trainings. The survey included questions on current fidelity monitoring methods at their agency, acceptability and feasibility of potential fidelity monitoring methods, and strategies for facilitating fidelity monitoring. Other items measuring EBP training and implementation were included in the survey, but only the survey items inquiring about fidelity monitoring were analyzed here.

#### Qualitative

BH clinicians, supervisors, and agency leaders in Indiana (hereafter referred to as BH partners) and leaders of IOs across the United States were recruited to participate in individual semi-structured interviews. Clinicians were selected for recruitment using convenience and snowball sampling methods.^38^ Eligible agency leaders were identified through review of staff directories on the websites of community mental health centers in Indiana. Eligible clinicians were selected from participant rosters for prior EBP trainings and through referrals by other participants. IOs were selected if they focused on supporting the dissemination, implementation, and fidelity monitoring of multiple EBPs. IOs that were housed in an academic context and/or had multiple streams of funding support were preferred. We expanded our IO recruitment to the national level, due to a lack of IOs in Indiana that fit these criteria and to ensure an adequate sample size and broad perspective on fidelity monitoring infrastructure. Eligible IO leaders were identified through review of selected IO websites and correspondence with IO contacts. Of 17 BH partners and 16 IO leaders who were invited, 10 BH partners and 11 IO leaders completed interviews. Participants received $100 in compensation. Interview questions were developed based on prior fidelity and implementation literature^11,12,39–42^ and included questions about current fidelity monitoring and facilitators, barriers, and priorities related to ongoing fidelity monitoring. Following the interview, participants were sent a brief survey to collect BH partner and IO leader demographics and organization information. The interviews were conducted remotely via secure videoconferencing software (Zoom) by the first and last authors. Interviews were recorded and transcribed.

### Survey measures

#### Provider demographics and practice characteristics form.^43,44^

A version of this form was completed by all participants. This form measured participants’ demographics (gender, age, race), professional background, and practice characteristics (primary role, highest degree, professional discipline, licensure status, years of experience in behavioral health field, current employment setting, years with current employer). IO leaders answered additional questions pertaining to the funding mechanism and size of their agency.

#### Fidelity monitoring questions

The fidelity monitoring questions were developed based on previous fidelity and implementation studies.^11,12,40,45–47^ Prior to the first question, a description of fidelity and examples of fidelity monitoring methods were provided: Fidelity is the extent to which an intervention is delivered as it was intended to be. Some examples of methods for monitoring fidelity have included observing and coding video- or audio-recorded therapy sessions; therapist self-report of what they did in a session or during treatment; patient report of what occurred in session; review of patient charts or treatment plans; and role-play assessments in which a therapist is given a scenario and asked to demonstrate a specific therapy skill or strategy.^11,12,40^ In the first question, participants were asked if their agency currently monitors what practices or therapies are being delivered (yes or no). Second, participants who indicated their agency monitors what practices or therapies are being delivered were asked to select which methods their agency uses. The third question asked participants to indicate which methods would be acceptable and feasible for monitoring fidelity at their agency. The final question asked participants to indicate which strategies they believed would facilitate fidelity monitoring in their practice. See Table 2 for the specific fidelity monitoring methods and potential facilitators asked about in this set of questions.

### Analyses

#### Quantitative data analysis

Descriptive data summarizing demographics, practice characteristics, and responses to the fidelity monitoring questions are presented in Tables 1 and 2. Participants who did not respond to the fidelity monitoring questions (*n* = 14) were excluded from analyses. Analyses were performed using SPSSv29.

**Table 1:** Indiana Behavioral Health Workforce survey participant demographic and practice characteristics (*N* = 191)

*Demographic and practice variables*	*N* (%)
*M* (*SD*), range years of age^a^	48.88 (11.93), 23 to 91
Gender^b^	
Female	150 (79.8%)
Gender queer	1 (0.5%)
Male	37 (19.7%)
Race	
American Indian or Alaskan Native	6 (3.1%)
Asian or Asian American	2 (1.0%)
Black or African American	24 (12.6%)
White	149 (78.0%)
Prefer not to say	10 (5.2%)
Hispanic, Latino/a/x, or Spanish Origin	4 (2.1%)
Professional discipline	
Social work	58 (30.4%)
Counseling	58 (30.4%)
Substance use/addiction	34 (17.8%)
Psychology	13 (6.8%)
N/A	8 (4.2%)
Nursing	6 (3.1%)
Other mental health related	4 (2.1%)
Other	4 (2.1%)
Legal/justice system related	3 (1.6%)
Marriage and family therapy	2 (1.0%)
Psychiatry	1 (0.5%)
Highest degree	
High school diploma or GED	11 (5.8%)
Some college	22 (11.5%)
Associate’s	13 (6.8%)
Bachelor’s	37 (19.4%)
Master’s	91 (47.6%)
Doctoral	12 (6.3%)
Specialist	4 (2.1%)
Other	1 (0.5%)
Mental health licensure status	
Trainee (e.g., student, intern)	11 (5.8%)
Post-Masters/Post-Doctorate (e.g., provisionally licensed)	9 (4.7%)
Licensed mental health provider	70 (36.6%)
Licensed or certified behavioral health provider (e.g., ICAADA, LCAC, LSW)	42 (22.0%)
Other	7 (3.7%)
N/A	52 (27.2%)
Employment setting	
Other (e.g., recovery center/substance use treatment clinics, hospital-based)^c^	58 (30.4%)
Outpatient or community mental health center	52 (27.2%)
Private practice	30 (15.7%)
Residential treatment facility or group home	28 (14.7%)
Jail or correctional facility	17 (8.9%)
HMO, PPO, or other managed care organization	10 (5.2%)
Day treatment or partial day hospital	6 (3.1%)
College or university	6 (3.1%)
Inpatient hospital or medical clinic	6 (3.1%)
Elementary, middle, high school	3 (1.6%)
*M* (*SD*), range years with current employer^d^	6.18 (7.45), 0–38
*M* (*SD*), range years in behavioral health field^e^	13.41 (11.39), 0–52
Primary role^a^	
Therapist/counselor	60 (31.4%)
Agency director or program manager	35 (18.3%)
Recovery coach/specialist	22 (11.5%)
Supervisor	13 (6.8%)
Case manager	12 (6.3%)
Executive leaders	7 (3.7%)
Quality assurance/training	7 (3.7%)
Nurse practitioner	2 (1.0%)
Psychiatrist	1 (0.5%)
Other	32 (16.8%)

*Note.* ^a^*N* = 4 were missing from this data; ^b^*N* = 3 were missing from this data; ^c^Most common “other” category was recovery center/substance use treatment clinics (*n* = 15); ^d^*N* = 3 were missing this data; ^e^*N* = 6 were missing this data

**Table 2:** Current fidelity monitoring practices (*N* = 191)

*Agency currently monitors fidelity*	*N* (%)
Yes	125 (65%)
No	66 (35%)
***Fidelity monitoring methods used***^*a*^	***N* (%)**
Therapist self-report	74 (59%)
Review of patient charts or treatment plans	74 (59%)
Review of billing codes	28 (22%)
Patient report	20 (16%)
Submitting video or audio recordings of therapy sessions	15 (12%)
Role-play assessments	9 (7%)
Live supervision or observation	4 (3%)
***Acceptable and feasible ways to monitor fidelity***	***N* (%)**
Review of patient charts or treatment plans	106 (55%)
Therapist self-report	102 (53%)
Patient report	58 (30%)
Role-play assessments	47 (24%)
Review of billing codes	36 (18%)
Submitting video or audio recordings of therapy sessions	30 (15%)
Live supervision or observation	3 (1%)
***Frequency at which fidelity monitoring should occur***^**b**^	***N* (%)**
Weekly	43 (22%)
Monthly	62 (32%)
Quarterly	53 (27%)
Every 6 months	23 (12%)
Yearly	9 (4%)
***Potential incentives to facilitate fidelity monitoring***^**b**^	***N* (%)**
Incentives for submitting session recordings	46 (24%)
Required for certification or rostering	78 (41%)
Required by employer	87 (45%)
Required by supervisor	55 (28%)
Administrative support	66 (34%)
Equipment resources	40 (21%)
Other	7 (3%)

*Note.* ^a^Only those who indicated their agency currently monitors what practices or therapies are being delivered completed this item (*N* = 125); ^b^*N* = 1 were missing this data

#### Qualitative data analysis

Given the timeline and need for rapid turnaround of results for the next phase of the service project, rapid qualitative analysis^48,49^ was selected to analyze the interviews. This strategy balances rigor with practical constraints and has been shown to be an effective, rigorous, and feasible alternative to traditional in-depth qualitative analysis.^48,50^ The qualitative analysis team consisted of two Doctoral-level, one Master’s-level researcher, and one undergraduate research assistant. First, a summary template was developed based on the interview questions. Next, the team tested the summary template on one transcript to identify any discrepancies, modify the template as needed, and clarify understanding. This iterative process was repeated on three transcripts until alignment and understanding was achieved.^51^ The remaining 17 transcripts were distributed among the team, with each transcript summarized by two team members. After each transcript was summarized using the summary template, consensus meetings were held to resolve any inconsistencies and discrepancies. Final transcript summaries were consolidated into a matrix. Coding team members then individually reviewed the matrix to generate a list of common themes and subsequently met together to collaboratively and iteratively review and then finalize the list of main themes.

#### Mixed methods analysis

The current study used a simultaneous approach to the collection and analysis of the quantitative and qualitative data (QUAN + QUAL).^52^ The function and purpose of the mixed methods analysis was to complement and connect the two methods, with both methods attempting to answer questions related to current fidelity monitoring practices in community BH care and for the qualitative interviews to offer greater understanding of the quantitative survey findings.

## Results

### Participants

#### Quantitative

A total of 205 participants completed the survey. Most participants (*n* = 191, 93.2%) completed the fidelity monitoring–related questions and are included in the current manuscript. Comparisons were conducted between completers and non-completers on demographics and practice information. Participants’ licensure status was significantly related to having completed the fidelity monitoring questions: *χ*^*2*^ (5, *N* = 205) = 13.49, *p* = 0.019. The percentage of participants who completed these questions was also significantly higher for those who identified as white (*p* = 0.04) and indicated working at outpatient or community mental health clinics (*p* = 0.02). Survey participants most frequently identified their primary role as therapist or counselor (*n* = 60, 31.4%), agency director or program manager (*n* = 35, 18.3%), and recovery coach or specialist (*n* = 22, 11.5%). The mean age of participants was 48.9 years old (*SD* = 11.93, range = 23 to 91), and they were predominantly female (*n* = 150, 79.8%), and white (*n* = 149, 78.0%). Almost half reported having a Master’s degree (*n* = 91, 47.6%), and the majority identified their professional discipline as either social work (*n* = 58, 30.4%) or counseling (*n* = 58, 30.4%). Diverse employment settings were endorsed, with the most common being outpatient or community mental health centers (*n* = 52, 27.2%), private practice (*n* = 30, 15.7%), and residential treatment facilities (*n* = 28, 14.7%). On average, participants reported their length of current employment as 6.2 years (*SD* = 7.45, range = 0 to 38) and 13.4 years (*SD* = 11.39, range = 0 to 52) of experience in the BH field. See Table 1 for participants’ demographic and practice characteristics.

#### Qualitative

Among the 10 BH partner interview participants, six were agency leaders (e.g., vice presidents or directors), two were managers or supervisors, and two were therapists. Participants were predominantly female (90%; male: 10%), white (90%; Black or African American: 10%), and had a Master’s degree (80%; Doctoral level: 20%). All participants reported working at outpatient or community mental health centers (100%). Participants on average were 43.6 years old (*SD* = 7.18, range = 29 to 52), had 15.4 years (*SD* = 9.25, range = 5 months to 24 years) of experience in the BH field, and had been at their current agency for 6.4 years (*SD* = 6.8, range = 5 months to 21 years).

All but one of the IO leaders reported being a director, with the one non-director indicating their role as vice president. All IO leaders were white (100%) and most had a Doctoral degree (63.6%; Master’s degree: 36.4%). Gender identity was not attained. IO leader participants on average were 50.3 years old (*SD* = 9.49, range = 36 to 70), had 24.1 years (*SD* = 10.8, range = 11 to 47) of experience in the BH field, and had been at their current employer for 15.0 years (*SD* = 10.8, range = 6 months to 42 years). The IOs were located in *N* = 11 unique states across the nation (West, *n* = 1; Southwest, *n* = 2, Southeast, *n* = 3; Northeast, *n* = 2; Midwest, *n* = 2). The IOs ranged in size from 3 to 50 full-time and part-time staff. IOs were primarily funded through state funding (e.g., State Mental Health Authorities, Departments of Mental Health, State Contracts), with additional federal funding (e.g., SAMHSA) or other contracts. Most IOs had an annual budget of over $1 million, with a range from $160,000 to $4.5 million per year.

### Mixed Methods Results Regarding Fidelity Monitoring

#### Current practices

Approximately 65% (*n* = 125) of survey respondents indicated their agency currently monitors what practices are being delivered (see Table 2 for a summary of the survey results). Similarly, the majority of those interviewed reported their agency (*n* = 9; 90%) or IO (*n* = 7; 63.6%) conducting some type of fidelity monitoring. Monitoring practices described during interviews with Indiana BH partners tended to be more intensive at the beginning of a clinician’s employment (e.g., in the initial few months) or dependent on the clinicians’ licensure status, with supervision as well as most monitoring occurring more frequently when clinicians were working towards licensure. While most IO leaders indicated their organization conducts fidelity monitoring, two leaders noted outsourcing fidelity monitoring to purveyor organizations or the state for certain EBPs (e.g., MINT, DBT). Three leaders reported their IOs did not currently monitor fidelity. See Table 3 for quantitative and qualitative questions and results.

**Table 3:** Quantitative and qualitative questions and results by participant group

Quantitative	Qualitative
**Conducts fidelity monitoring**	
*Q: Does your agency currently monitor what practices or therapies are being delivered in the agency?* · 65% (*n* = 125) indicated their agency current monitors what practices are being delivered	*BH Partners Q: Does your agency currently monitor what practices or therapies are being delivered in the agency?* · 90% (*n* = 9) reported their agency conducting some type of fidelity monitoring *IO Leaders Q: Does your Center currently conduct ongoing fidelity monitoring of the EBPs that have been implemented?* · 63.6% (*n* = 7) reported their agency conducting some type of fidelity monitoring
**Current fidelity monitoring practice(s)**	
*Q: How does your agency monitor what practices or therapies are being delivered? (mark all that apply)* · Therapist self-report: 59% · Chart/treatment plan review: 59% · Billing code review: 22% · Patient report: 16% · Reviewing recorded sessions: 12% · Role-play assessment: 7% · Direct observation/supervision: 3%	*BH Partners Q: How is this currently monitored?* · Direct observation (reverse shadowing, live/in-person observation): 70% · Chart/treatment plan review: 60% · Therapist self-report (supervision): 50% · Therapist self-report (checklist): 40% · Reviewing recorded sessions: 30% · Role-play assessment: 20% · Knowledge checks: 20% *IO Leaders Q: How is that monitored?* · Site visits: 27.3% · Reviewing recorded sessions: 27.3% · Direct observation/supervision: 18.2% · Coaching/consultation sessions: 9.1%
**Fidelity monitoring frequency**	
*Q: How often do you think fidelity monitoring should occur?* · Weekly: 22% · Monthly: 32% · Quarterly: 27% · Every 6 months: 12% · Yearly: 4%	Not asked
**Acceptable/feasible monitoring methods**	
*Q: What would be an acceptable and feasible way to monitor fidelity to a therapy or practice at your agency? (mark all that apply)* · Patient chart review: 55% · Therapist self-report: 53% · Patient report: 30% · Role-play assessment: 24% · Billing code review: 18% · Direct observation (recorded): 15% · Direct observation/supervision (live): 1%	*BH Partners Q: What would be an acceptable and feasible way to monitor fidelity to an EBP at your agency/in your practice?* · Preferred combination of methods · Observation · Supervision-related monitoring · Note audit/review *IO Leaders*: Not asked
**Barriers to fidelity monitoring**	
Not asked	*BH Partners:* Not asked *IO Leaders Q: Did your Center previously try to conduct fidelity monitoring data? If so, why did your Center decide to stop collecting fidelity monitoring data? Were there any barriers that contributed to your discontinuation of fidelity monitoring?* · Time- and resource-intensive · Limited existing infrastructure at agency- and/or state-level · Monitoring viewed as “auditing” · Potential consequences of low-fidelity scores · Not providing any incentives · Workforce shortage and turnover
**Barriers to recording sessions**	
Not asked	*BH Partners Q: What barriers do you think would interfere with clinicians/you being able to video or audio record therapy sessions at your agency?* · Agency policies · HIPAA/privacy-related concerns · Cost and equipment barriers · Clinician discomfort with feedback or evaluation *IO Leaders:* Not asked
**Facilitators to fidelity monitoring**	
*Q: Which of the following do you think could facilitate fidelity monitoring in your practice/agency? (check all that apply)* · Required by employer: 45% · Required for certification/rostering: 41% · Administrative support: 34% · Required by supervisor: 28% · Incentives for submitting recordings: 24% · Equipment/resources: 21% · Other: 3%	*BH Partners Q: What do you think could facilitate fidelity monitoring? Incentives for submitting recordings? If it was a requirement for a certification?* · Agency support/buy-in · Clinician motivation · Supportive infrastructure · Low burden fidelity monitoring methods · Monitoring viewed as a part of training IO Leaders: Is it incentivized?* · Agency support/buy-in · Clinician motivation · Supportive infrastructure · Offering incentives for fidelity monitoring · Feedback based on fidelity reviews · Strong relationships with clinicians · Partnering with an external entity

*Q*, Question; *EBP(s)*, evidence-based practice(s); *HIPPA*, Health Insurance Portability and Accountability Act; *Though not explicitly asked, discussion of barriers with IO leaders also illuminated facilitators

#### Fidelity monitoring methods

Among participants whose agencies engage in fidelity monitoring, clinician self-report (59%) and review of patient charts or treatment plans (59%) were the two most frequently used methods, followed by review of billing codes (22%), patient report (16%), session recordings (12%), and role-play assessments (7%). An additional method reported by 3% of survey participants was live/direct observation.

Survey and interview participants consistently endorsed using clinician self-report and chart review in their agencies. Interestingly, though endorsed by less than 5% of survey participants, 70% (*n* = 7) of BH partner interview participants endorsed using direct observation of therapy sessions. An additional method which emerged in the BH partner interviews was using knowledge checks, such as a short quiz assessing a foundational understanding of the EBP, as a method for fidelity monitoring. In their interviews, IO leaders noted that the fidelity monitoring method was driven by the implemented EBP, with the most common methods being site visit fidelity reviews, observational coding of recorded sessions, monitoring through coaching/consultation sessions, and supervisor observation or monitoring.

#### Acceptable and feasible methods

The most frequently used fidelity monitoring methods indicated by survey participants were also endorsed as the most acceptable and feasible. The feasibility and acceptability of methods reported by survey participants are as follows, in order of descending frequency: review of patient charts or treatment plans (55%), clinician self-report (53%), patient report (30%), role-play assessments (25%), review of billing codes (19%), and submitting session recordings (16%). In the BH partner interviews, participants often preferred a combination of methods over a single method. Additionally, direct observation, monitoring that can happen in the context of supervision, and note audit and reviews were specific methods more commonly noted as feasible and/or acceptable in the interviews.

#### Facilitators

Strategies that could facilitate fidelity monitoring indicated by survey participants were agency requirement (45%), certification requirement (41%), administrative support (35%), supervisor requirement (29%), incentives for session recordings (24%), and equipment resources (21%). Interviews with BH partners and IO leaders revealed both similar and different strategies that could facilitate fidelity monitoring. Common facilitators of fidelity monitoring reported by both BH partners and IO leaders were agency support, fidelity monitoring infrastructure, and clinician motivation. Both BH partners and IO leaders commonly identified agency understanding and support of fidelity monitoring and buy-in as a facilitator to fidelity monitoring. Regarding fidelity monitoring infrastructure, BH partner interviewees reported the need for adequate staffing, and time available to monitor fidelity, particularly for supervisors. One clinician highlighted the need for supervisors to have time: “I think they try, but we’re all so busy. The supervisors are so busy because they’re covering for clinicians who left the agency. And I think everybody is like playing catch up most of the time honestly.” Examples of infrastructure to facilitate fidelity provided by IO leader interviews included having IO staff available to conduct fidelity, efficient fidelity tools, and building internal capacity in agencies to conduct fidelity monitoring. Clinician motivation, such as to improve their skills, was also cited as a facilitator by both BH partners and IO interviewees.

Facilitators commonly indicated by only BH partners were low-burden methods and positioning fidelity monitoring as a part of training. Specifically, BH partner interviewees noted the importance of fidelity monitoring being integrated into existing technology (e.g., electronic health record) and the fidelity observation form being specific to an EBP model as facilitators to fidelity monitoring. Moreover, unique to the IO leader interviews, IO leaders reported tying incentives to fidelity monitoring, such as an enhanced rate for meeting certain fidelity scores, or consistent with survey findings, fidelity tied to certification or rostering. One IO leader stated:

And I think it’s incentivized when the practice is tied to a policy like it is here. So, the policy will say, you cannot have a contract to do the service if you score less than this. And we’re going to recognize you if you are high performers.

Additional facilitators that emerged in IO leader interviews included providing feedback based on fidelity reviews, strong relationships with clinicians, and outsourcing or partnering with the state or other organizations.

#### Barriers to fidelity monitoring and recording sessions

During the IO leader interviews, the most frequently reported barriers to fidelity monitoring were that it is time- and resource-intensive and the lack of existing fidelity monitoring infrastructure at the agency- and/or state-level. Other identified barriers included feeling of “auditing” agencies or clinicians when monitoring fidelity, potential consequences of low-fidelity scores (e.g., risk for lower reimbursement rate if score is not met), not providing any incentives, and workforce shortage and turnover. An IO leader described this feeling of “auditing” and potential unintended consequences of linking fidelity scores to reimbursement and other incentives:

The other problem is for fidelity reviews themselves because the policy is relying too much on that review finding… We’re trying to use the tool first and foremost as a quality improvement tool, but when you start knowing that there’s so much consequence to a team’s funding, there’s got to be some implicit or explicit bias that starts happening in the ratings where you’re trying to give them the benefit as much as you can to tip them over. But it’s compromising the integrity of the review at the same time, potentially. I think that’s where the reviewers start feeling like auditors and that’s not what we want it to be.

BH partner interviewees were not asked specifically about barriers to fidelity monitoring but were asked about barriers to recording therapy sessions. BH partner interviewees cited several barriers that they think interfere with clinicians being able to video or audio record therapy sessions at their agency. BH partner interviewees mentioned agency policies (e.g., strict policies, lengthy approval process), HIPAA and privacy-related concerns, and cost and equipment barriers as hindering the recording of sessions. For example, one agency leader noted:

So, the ongoing supervision and it’s usually by a supervisor that’s certified or qualified from outside the organization. They have to review your case work with a patient, which then compromises patient health information. There’s also like you have to have the perfect setup with a room for observation. That costs money and it takes up space. You have to have a place with video recordings of interactions with patients. So, there’s all a lot of pieces there.

Another barrier indicated by interviewees was clinician discomfort with feedback or evaluation sometimes being an impediment to recording. One agency leader explained:

And I think people might feel intimidated. I know I did when I first started when I was asking for permission for recordings, I didn’t want to do it in the first place, so there’s a barrier because staff are like, “I don't want to be recorded. I don't want this on record. What if I mess up?”

## Discussion

The current study used a mixed methods approach to better understand current fidelity monitoring practices in community behavioral health settings. To the best of our knowledge, this study is the first to characterize fidelity monitoring practices occurring within community behavioral health settings. Findings revealed significant variation in how often and how long monitoring occurs as well as the typical approach or methods used. Around two-thirds of BH workforce survey participants reported their agency currently monitors what practices are being delivered. Similarly, most of the IO leaders indicated their IO conducts fidelity monitoring. In both the survey and interviews, the methods for monitoring used varied, ranging from clinician self-report to review of patient charts to site visit fidelity reviews. More research in larger samples and community behavioral health settings across states is needed to further delineate current fidelity monitoring practices.

Several BH partners described their agency’s monitoring as being dependent on the treatment model or population. For example, Assertive Community Therapy (ACT), a comprehensive, team-based treatment, is best-fit for monitoring through site visits,^53^ while fidelity to an individual outpatient therapy, such as Motivational Interviewing, may be monitored through direct observation of therapy sessions.^54^ BH partners also described monitoring as being more intensive and occurring more frequently at the beginning of a clinician’s employment, and/or being dependent on the clinicians’ licensure status. It remains unclear how often monitoring occurs following the initial few months of new employment with an agency or once clinicians have achieved licensure and are typically no longer receiving ongoing clinical supervision. Notably, studies have demonstrated that ongoing supports (e.g., feedback, coaching) are needed to sustain clinicians’ skills.^55,56^ To maintain skills and ensure behavioral health EBPs are being delivered with fidelity over time, future research should inquire about how often monitoring occurs across clinician experience levels to identify gaps and areas where ongoing support for clinicians could be bolstered to achieve optimal fidelity monitoring and clinical outcomes.

Although direct observation of sessions is considered the gold standard for monitoring fidelity,^27^ most survey respondents did not report submission of session recordings as a method used by their agency or reported that this method would be feasible and/or acceptable at their agency. Interestingly, however, 70% of BH partners endorsed their agency using direct observation in interviews. This discrepancy and higher endorsement of direct observation in the interviews may reflect a sample of BH partners who are more interested and engaged in fidelity monitoring. In general, the low endorsement of direct observation in the survey is unsurprising given prior literature that has found this method to be resource-intensive and often impractical for community mental health settings.^28,29^ Further, while the observational coding or rating of therapy session recordings was not explored within the interviews, this has also been shown to be laden with challenges. For example, many EBP fidelity coding systems require extensive training to establish reliability,^57^ coding entire therapy sessions can be time-consuming, and contracting out the coding of sessions recordings to purveyor or IOs may be cost prohibitive. Future work should explore what fidelity monitoring tools and coding systems agencies that engage in direct observation are using to assess fidelity (e.g., validated fidelity tools for specific evidence-based treatments, in-house tools, structured vs. unstructured tools), who in the agency reviews or codes the recorded sessions, the training required to use the agency’s fidelity tools, and how feedback is provided and used within the agency. Moreover, artificial intelligence–based technologies (e.g., Lyssn.io), which use language learning and processing models to automate coding and generate session summaries, present one promising, scalable, cost-efficient method for coding and rating session recordings for fidelity that may alleviate the resource burden of fidelity monitoring in resource-constrained settings.^15,58–60^ Future research should evaluate the feasibility of this method within community behavioral health settings and how it might address existing barriers to human-based fidelity coding of therapy sessions.

Given the challenges to using direct observation, alternative approaches to monitoring fidelity were more commonly used among survey respondents and interviewees, specifically clinician self-report and review of patient charts or treatment plans. While these methods may be more practical, both methods have been found to overestimate EBP adherence.^34,61,62^ Behavioral role-play assessment is another less resource-intensive approach for monitoring fidelity that has been found to be comparable to direct observation.^34^ However, few endorsed their agencies’ use of this method. Further, only approximately one quarter indicated role-play assessment being acceptable and/or feasible. While work to evaluate practical methods for assessing fidelity, particularly for when session recordings are not feasible, has begun,^34,63^ understanding clinicians and agencies perceived acceptability and feasibility of alternative approaches is needed to increase quality monitoring within community behavioral health settings and to ensure alternative approaches developed are considered acceptable, feasible, and appropriate by those intended to use it.

Consistent with previous studies,^24,29,64^ qualitative and quantitative findings highlighted multi-level barriers and facilitators to fidelity monitoring. At the agency-level, agency support, being required by the agency, fidelity monitoring infrastructure (e.g., adequate staffing, time available), and agency policies around recording were cited as determinants. Strategies that increase agency support of fidelity monitoring and build a “fidelity culture” within the organization could facilitate fidelity monitoring.^24^ Fidelity culture refers to a workplace environment or organizational culture that values, supports, and integrates fidelity monitoring into clinical practice. For example, integrating fidelity monitoring into existing workflows, such as embedding it as a part of ongoing clinical supervision; allotting clinicians and supervisors with time to record, review, and code sessions; establishing clear agency policies that enable recording of sessions; and providing necessary recording equipment and software should be examined as strategies in future work.

A system-level facilitator noted by IO leaders was tying incentives to fidelity monitoring. Given fidelity monitoring is not reimbursed by payers or income-generating,^60^ providing incentives related to fidelity monitoring could increase organizations and clinicians’ motivation to engage in fidelity monitoring. For example, Ohio requires fidelity reviews of ACT teams be completed every 12 months by an independent entity to receive Medicaid reimbursement.^65^ Nevertheless, IO leaders who provided implementation support in states that tied fidelity scores to certain incentives (e.g., enhanced reimbursement rates) found this strategy to be a double-edged sword, with unintended consequences emerging, such as a lower reimbursement rate or impacts on funding if an agency or team did not meet a fidelity score. Further research is needed to explore the influence of system-level strategies, such as incentives, on fidelity monitoring. Regarding EBP-level facilitators, almost half of BH workforce survey participants indicated that fidelity monitoring being required for certification/rostering in an EBP as a facilitator. However, requiring it as part of certification or training requirements is likely not sufficient alone to support fidelity monitoring without addressing the system-, agency-, and clinician-level barriers to fidelity monitoring.^24,45^

Finally, corroborating prior studies,^24,60^ clinician-level determinants included clinician motivation, views on fidelity monitoring, clinician discomfort with feedback or evaluation, and perceived HIPAA and privacy-related concerns. This current study, among others, has highlighted clinician discomfort around performing skills in front of others or being evaluated as a fidelity monitoring challenge.^24,60,66^ Interestingly, Creed et al.^60^ found that while community mental health therapists and clinical leaders indicated concerns about how fidelity monitoring would impact clinician confidence, they also indicated it could aid in and enhance professional growth, supervision, and training. Clarification and transparency around the purpose of fidelity monitoring, the type of feedback that will be received, and how this feedback will be used may reduce apprehension or anxiety around fidelity monitoring. Further, interviewees identified the positioning of fidelity monitoring as part of training as a facilitator to clinicians’ engagement in fidelity monitoring. These findings emphasize the importance of creating a “fidelity culture” within agencies that consider and support fidelity as an essential part of delivering EBPs to promote fidelity monitoring and ease clinician discomfort.^24^

### Limitations

The authors also want to acknowledge the limitations of the study. Data were collected as part of a quality improvement service project, not a formal research study. Accordingly, the samples for both the survey and interviews were recruited as a convenience and snowball sample and thus may not be representative of the broader population of community-based behavioral health professionals and leaders. For instance, their self-selection into the study may reflect greater interest in and enthusiasm for fidelity monitoring than the typical clinician, and given the survey was distributed through multiple channels, tracking total contact and response rate was not feasible. Relatedly, the small interview sample size may limit the breadth of perspectives across the BH workforce. Further, all survey respondents were drawn from a single midwestern US state; thus, the process and results may not be representative of other states and territories—where workforce shortage and administrative burden may be less prevalent. Nevertheless, the inclusion of perspectives from multiple community partners (IO leaders, agency leaders, clinicians, supervisors, etc.) is a strength of this project.

There are also limitations arising from our measures and methods of analyses. First, despite its efficiency, and evidence as an acceptable alternative to traditional qualitative analysis,^48,50^ the use of rapid qualitative analysis may miss nuanced themes when compared with traditional analysis methods. Second, although the authors asked about fidelity in general and attempted to define fidelity (i.e., “Fidelity is the extent to which an intervention is delivered as it was intended to be”), the specific components of adherence (compliance with EBP protocol) and competence (skill level, or quality of delivery) were never specified, so the results may not map perfectly onto the existing literature. Third, our measures did not explicitly ask participants if their organization utilized EBPs and if so, which EBPs. As such, there may be variation in use of fidelity monitoring between organizations who utilize EBPs and those who do not. Fourth, survey and interview participants were not asked to specify which EBPs were being monitored at their agency. Finally, the authors collected individual reports of fidelity monitoring practices but were not able to collect an aggregate of reports from individuals within an agency, so the perspective of one person may not be reflective of everyone within the agency, potentially over- or underestimating fidelity monitoring practices.

## Implications for Behavioral Health

This study described current fidelity monitoring efforts and common determinants to fidelity monitoring among BH professionals in Indiana and within IOs across the United States. Overall, there was variability in fidelity monitoring practices and methods, with alternative approaches to fidelity monitoring, such as self-report and review of patient charts and treatment plans the most common, and the gold standard of direct observation infrequently used. Further, this study focused on EBPs in general, but future work should explore current fidelity monitoring practices for specific behavioral health EBPs (mental health vs. substance use disorder EBPs) and populations (e.g., youth vs. adult EBPs). Given the variability in monitoring practices and methods, future efforts aimed at increasing the uptake of fidelity monitoring within agencies should assess current fidelity monitoring practices through an internal audit or needs assessment to identify existing fidelity monitoring infrastructure and current gaps in monitoring. Results also identified common barriers and potential strategies for facilitating and supporting fidelity monitoring efforts. These findings emphasized the need for multi-level approaches that address system-, agency-, and clinician-level barriers. For example, at the system-level, state or federal agencies could implement policies to support and provide funding for agencies to develop monitoring practices and infrastructure as well as policies to incentivize ongoing monitoring. Agency-level strategies might include building a fidelity monitoring infrastructure, including having dedicated monitoring staff, and well-defined agency policies around recording. At the clinician-level, facilitative strategies could include having dedicated time towards monitoring built into clinicians’ schedules or supervision process and clear fidelity tools and feedback to alleviate potential clinician discomfort. These multi-level approaches will in turn build a “fidelity culture” within the agency, or a culture in which fidelity monitoring is considered and supported by the agency as an essential part of delivering EBPs, to successfully increase fidelity monitoring in community behavioral health settings.

## Data Availability

To protect the anonymity and confidentiality of study participants, no data will be available for public view.
